# Diet quality is associated with malnutrition and low calf circumference in Canadian long-term care residents

**DOI:** 10.1186/s40795-019-0314-7

**Published:** 2019-12-09

**Authors:** Natalie Carrier, Lita Villalon, Christina Lengyel, Susan E. Slaughter, Lisa Duizer, Jill Morrison-Koechl, Heather Keller

**Affiliations:** 1 École des sciences des aliments, de nutrition et d’études familiales, Faculté des sciences de la santé et des services communautaires, Université de Moncton, Moncton, NB E1A 3E9 Canada; 2 Faculty of Agricultural & Food Sciences, University of Manitoba, 35 Chancellor’s Circle, Winnipeg, MB R3T 2N2 Canada; 3 Faculty of Nursing, University of Alberta, Edmonton, AB T6G 1C9 Canada; 4 Department of Food Science, University of Guelph, Guelph, Ontario N1G 2W1 Canada; 5 Schlegel-University of Waterloo Research Institute for Aging, 250 Laurelwood Drive, Waterloo, ON N2J 0E2 Canada; 6 Applied Health Sciences, University of Waterloo, 200 University Ave W, Waterloo, ON N2L, 3G1 Canada

**Keywords:** Diet quality, Long-term care, Malnutrition, Calf circumference, Eating assistance

## Abstract

**Background:**

Older adults living in long-term care (LTC) are nutritionally vulnerable. The purpose of this study was to determine diet quality of Canadian LTC residents and its association with malnutrition and low calf circumference.

**Methods:**

A cross-sectional study was undertaken in 32 LTC homes across four Canadian provinces. Nutrient adequacy ratios (NARs) were calculated for seventeen nutrients; mean adequacy ratio (MAR) was calculated to describe overall diet quality. Malnutrition risk was assessed with the Mini Nutritional Assessment-Short Form (MNA-SF) and diagnosis of protein/energy malnutrition with the Patient-Generated Subjective Global Assessment (PG-SGA). Calf circumference (CC) was also assessed. Linear and logistic regressions for these outcomes with diet quality as the predictor were conducted adjusting for covariates.

**Results:**

Average MNA-SF score was 10.7 ± 2.5. Residents (43.5%) had mild/moderate to severe malnutrition based on the PG-SGA and 32.6% had a CC of < 31 cm. Mean MAR score was 0.79 ± 0.09 with significant differences between those requiring eating assistance (0.77 ± 0.11) and those that did not require assistance (0.80 ± 0.07) (*p* < .05). MAR score was significantly associated with malnutrition in fully adjusted models: MNA-SF scores [β = 5.34, 95% Confidence interval (CI) (2.81, 7.85)] and PG-SGA [Odds ratio (OR) = 0.49, 95% CI (0.38, 0.64)]. Those who had better diet quality were more likely to be well nourished or not at risk. Although several individual nutrients were associated with low CC (< 31 cm), there was no association between overall diet quality (MAR) and low CC.

**Conclusions:**

Diet quality is associated with malnutrition and individual nutrients (NARs) with a low CC. In addition to calories and protein, nutrient dense diets that promote adequate micronutrient intake are required in LTC.

## Background

Older adults living in long-term care (LTC) facilities are nutritionally vulnerable [1–3]. Poor food and fluid intake, resulting from multifactorial challenges such as dysphagia, is a primary cause for malnutrition in this setting [4]. Residents’ average energy intake is estimated at 1500 kcal/day or less [4–6]. With low energy intake, it is not surprising that up to 70% of residents have lower than recommended intakes of many micronutrients [4, 5, 7]. Diet quality is defined in this study as meeting protein and micronutrient requirements relative to the Recommended Dietary Allowance of the Dietary Reference Intakes. Identifying what specific nutrients are lacking in the diet is worthwhile as potential targets for intervention. It is anticipated that many of the sequelae of protein/energy malnutrition are also associated with specific micronutrient deficiencies and overall diet quality. A first step is to determine micronutrient inadequacies associated with protein/energy malnutrition.

Calf circumference (CC) is a simple tool that can be used as a surrogate indicator of muscle mass and as some have suggested, sarcopenia [8, 9]. A CC of < 31 cm indicates a risk of sarcopenia and frailty [10, 11]. To date, some studies have determined an association between vitamin D and loss of muscle function and strength in older adults [12–15], yet few studies have examined the associations between other micronutrients and muscle mass or low CC in this population.

Without careful menu planning and the use of standardized recipes and portion sizes, diet quality of menus can be poor [16]. Modified texture menus, especially those for pureed consumers, are typically lower in energy and micronutrients than regular menus [16–18]. Diet quality may also be further exacerbated for specific residents in LTC. For example, persons with dementia often require physical assistance with eating to improve their food and fluid intake [19–21]. Residents who lose the ability to eat are at a greater risk for malnutrition [22–24] and oral nutritional supplements (ONS) are often used to treat malnutrition [5, 24]. According to Simmons et al. (2010), offering residents a variety of foods and fluids in between meals may be a more effective way of improving nutritional status [25]. In the Making the Most of Mealtimes (M3) data set, we have demonstrated that about a third of micronutrients analyzed had high proportions (> 50%) of participants consuming levels below the recommendations [26]. However, limited research has explored the association between intake of specific nutrients or diet quality with malnutrition or low CC in LTC residents. This cross-sectional, multi-site study aimed to determine nutrient and overall diet quality of LTC residents and its association with malnutrition and low CC.

## Methods

### Study design and setting

This cross-sectional study was undertaken in 32 LTC homes across four Canadian provinces: Alberta, Manitoba, New Brunswick and Ontario and conducted between October 2014 and March 2016. The main study questions regarding determinants of food intake and prevalence of inadequate intake were used to determine the sample size. In brief, a convenience sample of homes was recruited in each province to promote diversity (e.g., profit structure, corporate vs. independent homes, ethnic focus, size). Home eligibility criteria included: being in operation for at least six months; and having a minimum of 50 residents who met the resident eligibility criteria. One to four units in each home were randomly selected for recruitment of participants; if the home had a dementia care unit, this was included.

### Participants

Residents were randomly selected and recruited to reach a quota of 20 residents per home. Participant inclusion criteria were: 65+ years of age; not palliative and medically stable (no hospital admission in previous month); not recently or temporarily admitted to the home; not requiring tube feeding or end of life care; and mostly eating in the dining room. Informed written consent was provided by the resident or their alternate decision-maker. The resident sample size per province (*n* = 160) and for the entire study (*n* = 640) was based on the analyses planned for the main study (multi-level regression modeling) [27], which allowed identification of the independent determinants of inadequate food intake. One participant revoked their consent to participate after data were collected, bringing the total sample to 639 participants. Of these, only 619 participants had complete data on all variables of interest for this paper.

### Data collection tools and procedures

Four trained (dietetic program prepared or dietitian) research coordinators collected health and nutritional status data. Details on all data collected can be found in the protocol paper [27] and only those variables used in this analysis will be described. Resident data, such as diagnosis, prescription of ONS, and dietary prescription were collected from health charts. In addition, modified texture diets (MTD) were classified for analysis using the International Dysphagia Diet Standardisation Initiative (IDDSI) [16, 28] as more than 60 diet textures were represented in the data. Specifically, five categories of the IDDSI continuum were used to categorize food texture (3 = liquidized, 4 = pureed, 5 = minced and moist, 6 = soft and bite-sized, and 7 = regular texture). Modified texture diets were dichotomized as either yes or no; “yes” representing categories 3, 4 and 5 of the IDDSI and “no” representing categories 6 and 7. Activities of daily living and cognitive performance were collected using the interRAI-LTCF 2.0 [27–29]. The interRAI-LTCF provides a standardized and validated means of collecting comprehensive clinical information on LTC residents [29]. Residents’ CC was measured using standardized procedures [30]. Health record information and discussion with staff, family and/or the resident were used to complete the Mini Nutritional Assessment-Short Form (MNA-SF) [31] and the resident was examined to complete the Patient-Generated Subjective Global Assessment (PG-SGA) [32]. The MNA-SF scores were continuous, ranging from 0 to 14, where higher scores indicated better nutritional status and a cut-point of <12 indicated malnutrition risk. The PG-SGA ratings of A (well nourished), B (mild/moderate malnutrition) and C (severe malnutrition) were used; the numerical score was not used as it has not been validated for this population [27]. The PG-SGA ratings were dichotomized such that mild/moderate and severe malnutrition were combined (B/C) for this analysis.

### Dietary assessment

Two trained research assistants per province collected meal-level data for each participant. Researchers completed a standardized form three times (once per day of food intake data collection) to identify eating behaviours and mealtime experiences of participants. Physical assistance with eating was determined by observation using a single item from the validated Edinburgh Feeding Evaluation in Dementia Questionnaire (Ed-FED-Q) and nine additional eating challenges (e.g., does the resident get distracted, do they cough during the meal, choke etc.) were also recorded and scaled to be consistent with Ed-FED-Q (never (1), sometimes (2), frequent (3)) [33]. The rounded average of these three mealtime observations were used and residents categorized as no physical assistance vs. requiring physical assistance. Three non-consecutive days (two weekdays and one weekend day) of weighed food intake (before and after consumption of main plates), with estimated beverages, side dishes and snacks, were collected for each resident. Nutritional analysis software (ESHA Food Processor, version 10.14.1), with the Canadian Nutrient File, was used to obtain mean daily intake of energy (kcal), protein (g), and several micronutrients. Oral nutritional supplements to promote energy and protein intake either at meals, snacks or medical rounds were included in this analysis. Micronutrient supplement use was not included in this analysis; consumption of vitamins and minerals is based on intake of food, beverages and any ONS. For this study, diet quality was determined by nutrient adequacy ratios (NARs) for seventeen nutrients (calcium, copper, folate, iron, magnesium, phosphorus, selenium, vitamins A, B_1_, B_2_, B_3_, B_6_, B_12_, C, D, E [α-tocopherol], zinc) and a mean adequacy ratio (MAR), which was the average of the seventeen NARs. NARs were calculated as the proportion consumed relative to the corresponding sex and age-specific. Recommended Dietary Allowance for each nutrient; a maximum of 1.0 indicated that the recommendation was met/exceeded [34] and a MAR score closer to 1.0 indicated better overall diet quality. All data were collected on paper forms and transferred to RedCAP (Vanderbilt University) for transfer to the research centre for analysis.

### Statistical analyses

Descriptive statistics (mean, median, frequency) of resident characteristics were computed. The associations of each diet parameter (i.e., energy and protein intake, MAR, NARs) with nutritional risk, malnutrition and low CC, were tested. Models were adjusted for age and sex as these demographic variables drive food intake. Hierarchical regression analysis accounted for clustering at the home and unit levels; linear regression tested the association with MNA-SF score while logistic regression estimated the odds ratios for PG-SGA risk and low CC. In addition to the simple models adjusted for age and sex, fully adjusted models also included three resident-level covariates (i.e., MTD (IDDSI categories 3–5), ONS use, requiring physical assistance (sometimes or often)) that are prevalent in this sample and demonstrated to be associated with malnutrition [5, 16–25]. Statistical significance was set at *p* < 0.05. All analyses were performed using SAS/STAT® 9.4 statistical software (SAS Institute Inc., Cary, North Carolina). This study was approved by the research ethics boards at the University of Waterloo (ORE#20056), University of Alberta (Pro00050002), University of Manitoba (J2014:139), and Université de Moncton (1415-022) and complies with the current laws of Canada.

## Results

The sample is described in Table 1. Average age of participants was 86.8 ± 7.8 years, 31.2% were men, and 55.5% had moderate to severe cognitive impairment. Almost a quarter of participants required eating assistance, one-third were on MTD, and one-third were prescribed ONS. Mean CC was 33.3 ± 4.8 with 32.6% having a CC of < 31 cm. Mean MNA-SF score was 10.7 ± 2.5, with 53.3% below the malnutrition risk cut-point (< 12) while 43.5% were classified as malnourished with PG-SGA.

**Table 1:** Sample characteristic and outcomes of interest

Resident Characteristics	Mean (SD)
Age, years	86.8 (7.8)
Sex, male (%)	31.2
Moderate/severe cognitive impairment, (CPS^a^ score ≥ 3), yes (%)	55.5
Ed-FED-Q Score^a^	12.4 (2.3)
Other eating behaviors Score (maximum = 27)^a^	10.6 (1.6)
Activity of daily living Scale^a^	15.0 (7.8)
Physical assistance (sometimes or often) with eating required^a^ (%)	22.9
Total number of diagnoses	5.4 (2.0)
Any ONS prescribed, yes (%)	30.5
MTD (IDDSI category 3–5), yes (%)	33.0
Outcome variables	
CC, cm	33.3 (4.8)
CC < 31cm^b^ (%)	32.6
MNA-SF	10.7 (2.5)
Malnourished/At risk of malnutrition (MNA-SF < 12) (%)	53.3
PG-SGA (Malnutrition = B/C) (%)	43.5

^a^Missing values; Ed-FED-Q, *n* = 616; Other eating behaviors score, *n* = 616; Activity of daily living score, *n* = 614; Physical Assistance, *n* = 616

^b^As per Mini Nutritional Assessment cut-point (MNA) [31]

Residents’ food intake and diet quality by MTD use, ONS prescription and physical assistance with eating are provided in Table 2. Participants’ mean adjusted energy intake was 1556.3 ± 294.1 kcal/day and protein intake was 57.5 ± 13.0 g/day. Almost two-thirds of participants were meeting the required daily intake of protein using the 0.8 g/kg body weight/day cut-point. Participants using MTD or ONS or those that required any physical assistance with eating had significantly lower energy intake than those on regular texture diets, not prescribed ONS or not requiring assistance with eating. Protein intake was only significantly lower in participants requiring assistance with eating as compared to those not requiring any eating assistance (54.9 ± 14.7 vs. 58.4 ± 12.2). Mean MAR score (diet quality) for the total sample was 0.79 ± 0.09 with those requiring assistance with eating having significantly lower scores than participants not requiring physical assistance with eating. No significant difference in MAR were observed between participants on regular texture diets and those on MTD and between participants prescribed ONS and those that were not. For the total sample, the mean NAR scores were greater than 0.70 for the majority of the 17 nutrients (maximum of 1.0 indicating that Recommended Dietary Allowance for the nutrient was met or exceeded) except for the following five nutrients, which had much lower NAR scores: vitamin D (0.28), vitamin E (0.34), folate (0.58), calcium (0.61) and magnesium (0.65). Individual NAR scores for most nutrients were consistently lower in participants on MTD, ONS, and requiring physical assistance with eating, but for a few exceptions. Interestingly, NAR score for vitamin C was significantly higher in MTD, individuals prescribed ONS, and those requiring physical assistance with eating. Scores for vitamin D and calcium were significantly higher in participants on MTD and the NAR score for vitamin E was significantly higher in participants prescribed ONS.

**Table 2:** Food intake and diet quality by diet texture, oral nutritional supplements and assistance with eating

	Total Sample	Modified Texture Diet (IDDSI 3–5) Mean (SD)		Oral Nutritional Supplements Prescribed Mean (SD)		Physical Assistance with Eating Sometimes/Often^a^ Mean (SD)	
		No	Yes	No	Yes	No	Yes
Number of Residents (n)	619	415	204	430	189	475	141
Energy Intake (kcal/day)	1556.3 (294.1)	1578.9 (274.3)	1510.2 (326.7)*	1580.3 (272.6)	1501.6 (332.4)*	1583.1 (276.6)	1478.0 (323.1)*
Protein intake (g/day)	57.5 (13.0)	57.5 (11.7)	57.5 (15.3)	58.0 (12.1)	56.3 (14.8)	58.4 (12.2)	54.9 (14.7)*
MAR	0.79 (0.09)	0.80 (0.08)	0.78 (0.10)	0.79 (0.07)	0.78 (0.11)	0.80 (0.07)	0.77* (0.11)
NAR							
Vitamin D^b^	0.28 (0.11)	0.27 (0.11)	0.30* (0.12)	0.27 (0.11)	0.28 (0.12)	0.28 (0.11)	0.27 (0.12)
Vitamin E α-tocopherol	0.34 (0.13)	0.34 (0.13)	0.34 (0.14)	0.31 (0.09)	0.40* (0.19)	0.34 (0.13)	0.35 (0.14)
Folate (DFE)	0.58 (0.18)	0.63 (0.17)	0.48* (0.17)	0.62 (0.16)	0.51* (0.21)	0.61 (0.19)	0.48* (0.19)
Calcium	0.61 (0.19)	0.60 (0.18)	0.64* (0.20)	0.61 (0.18)	0.62 (0.20)	0.62 (0.19)	0.60 (0.19)
Magnesium	0.65 (0.15)	0.65 (0.14)	0.64 (0.17)	0.64 (0.13)	0.66 (0.17)	0.66 (0.13)	0.62* (0.17)
Vitamin B_6_	0.75 (0.16)	0.74 (0.15)	0.75 (0.18)	0.74 (0.15)	0.76 (0.19)	0.75 (0.15)	0.74 (0.19)
Zinc	0.81 (0.16)	0.80 (0.16)	0.82 (0.18)	0.80 (0.15)	0.82 (0.18)	0.82 (0.15)	0.79 (0.18)
Vitamin A (RAE)	0.82 (0.17)	0.82 (0.16)	0.81 (0.18)	0.83 (0.16)	0.78* (0.19)	0.82 (0.16)	0.79 (0.20)
Vitamin C	0.90 (0.17)	0.88 (0.19)	0.94* (0.13)	0.89 (0.18)	0.94* (0.15)	0.89 (0.18)	0.95* (0.14)
Copper	0.92 (0.12)	0.93 (0.11)	0.91 (0.15)	0.92 (0.12)	0.92 (0.14)	0.94 (0.11)	0.88* (0.16)
Vitamin B_1_	0.93 (0.13)	0.95 (0.11)	0.90* (0.16)	0.95 (0.10)	0.90* (0.16)	0.95 (0.10)	0.89* (0.17)
Selenium	0.96 (0.12)	0.99 (0.06)	0.92* (0.18)	0.98 (0.07)	0.92* (0.17)	0.98 (0.06)	0.90* (0.19)
Iron	0.97 (0.09)	0.98 (0.07)	0.95* (0.12)	0.97 (0.08)	0.95* (0.11)	0.98 (0.06)	0.94* (0.12)
Vitamin B_12_	0.97 (0.08)	0.98 (0.08)	0.96 (0.10)	0.97 (0.08)	0.96 (0.09)	0.98 (0.07)	0.96* (0.11)
Vitamin B_2_	0.98 (0.07)	0.99 (0.04)	0.97* (0.10)	0.99 (0.05)	0.97* (0.09)	0.99 (0.04)	0.96* (0.11)
Vitamin B_3_^c^	0.98 (0.07)	0.99 (0.04)	0.96* (0.12)	0.99 (0.04)	0.96* (0.11)	0.99 (0.04)	0.96* (0.12)
Phosphorus	0.98 (0.06)	0.99* (0.05)	0.98* (0.08)	0.99 (0.04)	0.97* (0.09)	0.99 (0.04)	0.97* (0.09)

^a^Data missing for 3 residents, *n* = 616. Food and nutrient intake stratified by modified texture diet, oral nutritional supplement use and requiring physical assistance, as these resident level covariates were a) prevalent in the sample, and b) known to be associated with malnutrition

^b^International unit

^c^Niacin equivalent

*p < 0.05

Diet parameters and their association with nutrition risk (MNA-SF), malnutrition (PG-SGA) and low CC adjusted for home, unit, age and sex (simple model) are presented in Table 3. Median MAR score for the total sample was 0.80 [interquartile range (IQR) = 0.75, 0.85] and vitamins D, E, folate and calcium had the lowest median NAR values. The full models adjusted for MTD, ONS prescription, and physical assistance with eating are presented in Table 4. In Tables 3 and 4, a positive parameter estimate (β > 0) indicates a higher MNA-SF score (i.e., better nutrition) and an odds ratio less than 1 indicates decreased risk of malnutrition (PG-SGA) and a lower likelihood of low CC. The MAR was positively associated with MNA-SF scores in both adjusted models, indicating that higher diet quality is associated with better nutrition. Likewise, a higher MAR score was associated with lower odds of malnutrition (PG-SGA), even when fully adjusted [OR = 0.42; 95% CI (0.31, 0.58); Table 4], but was not associated with a lower CC in either model. The NAR scores for most nutrients (13 of the 17) had a significant positive association with MNA-SF score in the simple model (Table 3). In the fully adjusted models, ten nutrients remained significantly associated with MNA-SF score. Only one nutrient (vitamin C) had a significant negative association with MNA-SF score [β = − 1.37; 95% CI (− 2.58, − 0.17)], indicating that the higher vitamin C intake, the greater the risk of malnutrition, but this association was not significant in the fully adjusted model (Table 4). Higher NAR for all but two nutrients (vitamins C and E) were associated with a lower likelihood of being malnourished (PG-SGA) in the simple model (all OR < 1.0), and only vitamin B_3_ lost significance in the fully adjusted model. Examining low CC as the outcome, higher NAR scores for six of the key nutrients (folate, iron, magnesium, selenium and vitamins B_1_ and B_3_) were associated with reduced risk of low CC, whereas a higher vitamin E NAR [OR = 1.28; 95% CI (1.11, 1.49)] was associated with a greater risk of low CC in the simple model. Only magnesium was significantly associated with low CC [OR = 0.80; 95% CI (0.69, 0.94)] in the fully adjusted model; odds of low CC were reduced when the NAR for magnesium was higher.

**Table 3:** Association of diet quality with malnutrition risk and low calf circumference (simple model)

	Descriptive statistics	Linear Regression	Logistic Regression^a, b^	
Diet Quality Parameters	MAR/NAR^c^	MNA-SF	PG-SGA risk (score of B/C)	CC < 31
	Median (IQR)	Parameter Estimate (95% CI)	Odds Ratio^d^ (95% CI)	Odds Ratio (95% CI)
Energy Intake (kcal/day)	1551.8 (1375.0, 1742.0)	0.002 (0.001, 0.003)*	0.78 (0.72, 0.84)*	0.85 (0.78, 0.91)*
Protein intake (g/day)	56.5 (49.9, 65.1)	0.04 (0.02, 0.06)*	0.66 (0.55, 0.78)*	0.81 (0.68, 0.96)*
MAR	0.80 (0.75, 0.85)	5.34 (2.81, 7.85)*	0.49 (0.38, 0.64)*	0.80 (0.64, 1.00)
NAR				
Calcium	0.59 (0.46, 0.76)	1.42 (0.16, 2.68)*	0.83 (0.74, 0.93)*	0.94 (0.84, 1.06)
Copper	1.00 (0.88, 1.00)	3.55 (1.69, 5.42)*	0.68 (0.57, 0.82)*	0.88 (0.75, 1.03)
Folate (DFE)	0.58 (0.46, 0.70)	4.45 (3.24, 5.65)*	0.63 (0.56, 0.71)*	0.77 (0.68, 0.86)*
Iron	1.00 (1.00, 1.00)	5.52 (3.26, 7.78)*	0.48 (0.36, 0.65)*	0.74 (0.60, 0.92)*
Magnesium	0.64 (0.56, 0.74)	2.59 (1.03, 4.14)*	0.72 (0.62, 0.83)*	0.84 (0.73, 0.96)*
Phosphorus	1.00 (1.00, 1.00)	7.62 (4.32, 10.92)*	0.46 (0.30, 0.70)*	0.79 (0.59, 1.07)
Selenium	1.00 (1.00, 1.00)	4.88 (3.13, 6.62)*	0.46 (0.34, 0.62)*	0.73 (0.61, 0.87)*
Vitamin A (RAE)	0.84 (0.70, 0.99)	2.29 (1.01, 3.58)*	0.73 (0.64, 0.82)*	0.92 (0.81, 1.03)
Vitamin B_1_	1.00 (0.92, 1.00)	4.22 (2.55, 5.89)*	0.59 (0.49, 0.71)*	0.76 (0.66, 0.89)*
Vitamin B_2_	1.00 (1.00, 1.00)	5.56 (2.57, 8.56)*	0.49 (0.33, 0.73)*	0.90 (0.69, 1.17)
Vitamin B_3_^e^	1.00 (1.00, 1.00)	5.56 (2.77, 8.35)*	0.43 (0.26, 0.70)*	0.62 (0.46, 0.84)*
Vitamin B_6_	0.75 (0.63, 0.87)	0.86 (−0.54, 2.25)	0.80 (0.70, 0.90)*	0.98 (0.87, 1.11)
Vitamin B_12_	1.00 (1.00, 1.00)	3.18 (0.75, 5.62)*	0.64 (0.50, 0.82)*	1.06 (0.84, 1.32)
Vitamin C	1.00 (0.87, 1.00)	−1.37 (−2.58, −0.17)*	1.07 (0.96, 1.19)	1.09 (0.97, 1.22)
Vitamin D^f^	0.26 (0.19, 0.35)	0.85 (−1.28, 2.97)	0.80 (0.66, 0.96)*	0.97 (0.80, 1.18)
Vitamin E α-tocopherol	0.31 (0.26, 0.39)	−1.22 (− 2.88, 0.44)	1.12 (0.97, 1.30)	1.28 (1.11, 1.49)*
Zinc	0.82 (0.70, 0.95)	2.11 (0.68, 3.54)*	0.76 (0.66, 0.86)*	0.92 (0.81, 1.04)

^a^Logistic regression models did not converge when unit was included; adjusted for home

^b^Odds ratios calculated for: energy intake, per 100 kcal increase; protein intake, per 10g increase; energy/body weight, per 10 kcal/kg increase; protein/body weight, per 0.1 g/kg increase; adequacy ratio (MAR/NAR) scores are per 0.1 unit increase: < 1 lowers odds of the outcome, 1 = no association, and > 1 means higher odds of the outcome

^c^Proportion consumed relative to the Recommended Dietary Allowance for a given nutrient (NAR) or average of all nutrients (MAR), to a maximum of 1.0, which indicates the Recommended Dietary Allowance was met or exceeded

^d^Odds ratio adjusted for home, age, sex

^e^Niacin equivalent

^f^International unit

* *P* < 0.05

**Table 4:** Association of diet quality with malnutrition risk and low calf circumference (fully adjusted models)

	Linear Regression	Logistic Regression^a.b^	
Diet Quality Parameters	MNA-SF	PG-SGA risk (score of B/C)	CC < 31
	Parameter Estimate (95% CI)	Odds Ratio^c^ (95% CI)	Odds Ratio (95% CI)
Energy Intake (kcal/day)	0.001 (0.001, 0.002)*	0.77 (0.70, 0.85)*	0.87 (0.80, 0.95)*
Protein intake (g/day)	0.03 (0.01, 0.04)*	0.63 (0.51, 0.77)*	0.84 (0.70, 1.01)
MAR	3.58 (1.35, 5.82)*	0.42 (0.31, 0.58)*	0.86 (0.66, 1.11)
NAR			
Calcium	1.83 (0.75, 2.91)*	0.74 (0.64, 0.84)*	0.90 (0.80, 1.02)
Copper	2.12 (0.46, 3.78)*	0.71 (0.57, 0.87)*	0.94 (0.78, 1.14)
Folate (DFE)	1.68 (0.51, 2.85)*	0.74 (0.65, 0.85)*	0.93 (0.82, 1.06)
Iron	3.06 (0.92, 5.20)*	0.53 (0.38, 0.74)*	0.85 (0.67, 1.09)
Magnesium	2.46 (1.12, 3.81)*	0.63 (0.53, 0.75)*	0.80 (0.69, 0.94)*
Phosphorus	4.13 (1.15, 7.11)*	0.50 (0.32, 0.80)*	1.02 (0.72, 1.43)
Selenium	0.71 (−0.98, 2.39)	0.62 (0.43, 0.89)*	0.96 (0.79, 1.17)
Vitamin A (RAE)	1.32 (0.20, 2.45)*	0.70 (0.61, 0.81)*	0.97 (0.85, 1.11)
Vitamin B_1_	1.77 (0.24, 3.29)*	0.68 (0.55, 0.83)*	0.89 (0.75, 1.05)
Vitamin B_2_	1.76 (−0.94, 4.46)	0.55 (0.35, 0.86)*	1.18 (0.87, 1.60)
Vitamin B_3_^d^	0.82 (−1.77, 3.40)	0.68 (0.40, 1.15)	0.86 (0.63, 1.17)
Vitamin B_6_	1.14 (−0.07, 2.34)	0.69 (0.60, 0.81)*	0.95 (0.83, 1.09)
Vitamin B_12_	1.87 (−0.24, 3.99)	0.59 (0.44, 0.79)*	1.18 (0.91, 1.53)
Vitamin C	0.04 (−1.02, 1.10)	0.92 (0.81, 1.03)	0.97 (0.86, 1.10)
Vitamin D^e^	2.08 (0.25, 3.91)*	0.61 (0.49, 0.77)*	0.86 (0.69, 1.06)
Vitamin E α-tocopherol	0.90 (−0.59, 2.38)	0.84 (0.71, 1.01)	1.07 (0.91, 1.26)
Zinc	2.16 (0.92, 3.39)*	0.65 (0.55, 0.76)*	0.88 (0.76, 1.01)

^a^Logistic regression models did not converge when unit was included; adjusted for home only

^b^Odds ratios calculated for: energy intake, per 100 kcal increase; protein intake, per 10g increase; energy/body weight, per 10 kcal/kg increase; protein/body weight, per 0.1 g/kg increase; adequacy ratio (MAR/NAR) scores are per 0.1 unit increase: < 1 being lower odds of the outcome, 1 = no association, and > 1 meaning higher odds of the outcome

^c^Odds ratios adjusted for resident level characteristics that were prevalent and known to be associated with malnutrition (MTD, ONS, requiring physical assistance)

^d^Niacin equivalent

^e^International unit

* *P* < 0.05

## Discussion

This study examined diet quality of LTC residents using the MAR method and its association with malnutrition and low CC. Comparable to other studies conducted in LTC, approximately half of residents were malnourished or at risk of malnutrition according to the PG-SGA and MNA-SF [1–3]. The percentage of residents found to have low CC was similar to the percentage of residents at risk for malnutrition; CC is highly associated with malnutrition, as demonstrated in prior analyses of this data set [35].

Diet quality as assessed by MAR and several NARs was associated with lower risk of malnutrition when adjusting for ONS, MTD and eating assistance, confirming the importance of a nutrient dense diet in LTC facilities. As well, this association confirms that MNA-SF and PG-SGA, although focused on protein/energy malnutrition, also reflect micronutrient intake. However, no significant association was observed between MAR score and low CC and only the NAR for magnesium was significant in the fully adjusted model for low CC; protein in g/kg body weight was of borderline significance. This suggests that factors (e.g., eating challenges), other than micronutrients and diet quality may be more influential on low muscle mass.

Previous studies have found a positive association between mealtime assistance on energy intake of residents with dementia [36, 37]. Yet, the amount of eating assistance is critical. In the main analysis for M3, it was identified that those who received eating assistance ‘often’, had statistically significantly higher energy intake than those who received eating assistance ‘sometimes’ [38]. It was concluded that when one-on-one assistance is provided, residents’ needs are met, but when residents still participate in some independent eating they are at increased risk for low intake. In the current analysis, ‘sometimes’ and ‘often’ receiving eating assistance were amalgamated to provide a sufficiently large group for comparison to those who received no eating assistance. This likely explains the divergence in findings from the main analysis and that from prior research [26]. Further, as noted in prior M3 analysis, persons requiring eating assistance are commonly prescribed MTDs, which are often lower in key nutrients as compared to regular texture diets [16]. Few studies have yet to examine the influence of eating assistance on protein intake and micronutrient intake in LTC residents [38].

As expected, results show that higher energy intake was associated with higher MNA-SF score, indicating less risk of malnutrition and with lower odds of malnutrition and low CC. One study also found that residents with malnutrition or at risk thereof had lower energy intake [39]. Likewise, protein intake was associated with all three outcome measures. This corroborates the contention that low intake and not excess metabolic demand (as is seen as well in acute care malnutrition, surgery etc.) is a primary mechanism for malnutrition in LTC residents. Investigation into why low intake occurs using a comprehensive conceptual model helped to identify these relevant factors that impair food intake in the main analysis of this study [18, 38].

Mean NAR score for the total sample was low for five of the 17 micronutrients: vitamin D, vitamin E, folate, calcium, and magnesium, which corresponds to previous findings that show inadequate intakes from food/beverages for these nutrients in LTC [4, 5, 40]. However, it is important to note that vitamin E specifically is under-represented in nutrient analysis databases [41] due to poor reporting of this nutrient by manufacturers. The present study found that residents on MTD, on ONS or requiring physical assistance with eating were more likely to have lower intakes of nutrients than their comparators. In fact, ten of the 17 nutrients were significantly lower in residents requiring assistance with eating than those without eating assistance, nine nutrients were significantly lower in residents on ONS and eight nutrients were lower in residents on MTD. The only nutrient with a NAR higher for those requiring eating assistance was vitamin C. This may be due to increased use of MTD or ONS or some other covariate not modeled. Analysis to determine the percentage of residents on MTD and requiring eating assistance that are prescribed ONS should be further investigated. Those on MTD had a significantly higher NAR score for vitamin D (0.30), calcium (0.64) and vitamin C (0.94) than regular texture consumers. Food fortification, especially with vitamin D and calcium, has been shown to increase intake of fortified nutrients in LTC [42–44]. A prior analysis of this dataset demonstrates that recipe standardization and enhancement in some provinces improved nutrient density for MTD and specifically for these nutrients [16]. Few nutrients were lower for ONS consumers than non-consumers, yet vitamins E and C were higher in users than nonusers. As noted above, discrepancies in vitamin E content of food databases may explain this difference as all ONS include vitamin E and amounts are provided on labels.

Limitations to this work include the purposive sampling of homes, which likely do not represent all homes in Canada; therefore, generalization of the findings is cautioned. Random selection of homes was not feasible, but diversity was attained by recruiting homes with key characteristics such as culture, size, non/for profit, etc. By randomly selecting units and participants, selection bias was reduced; comparison of participants to the eligible pool in the home demonstrated that participants were representative of their home [27]. Data from this study suggests that some clinical improvements are required to prevent malnutrition and loss of muscle mass in LTC residents, such as: 1) improving nutritional intake of residents requiring texture-modified meals by the creation of nutrient dense, appealing and tasty foods to bring more pleasure to mealtimes; 2) providing quality and constant eating assistance during mealtimes to all residents who require physical support by training current LTC staff; and 3) ensuring nutrient-rich meals are provided by giving more attention to specific micronutrient content (i.e., vitamin D, vitamin E, calcium, folate, and magnesium) during menu planning.

## Conclusions

This analysis demonstrates that when attempting to prevent or mitigate malnutrition, attention to micronutrient intake, in addition to energy and protein, is needed. In the past, Canada’s Food Guide has been used to plan menus, potentially leading to inadequate micronutrient content [16]. The new Guide does not specify servings to be consumed per day and as a result the Dietary Reference Intake is recommended for menu development to avoid micronutrient deficiency [45]. Enhancement of the diet for protein and energy are relatively common practices in LTC [46], but micronutrient enhancement or fortification has been nominally studied or used in practice [46, 47]. Development of enhanced recipes that go beyond energy and protein are needed to support improved nutritional status of residents. This study differs from previous research, which has been limited to single sites/regions/provinces, by providing a pan-Canadian understanding of diet quality and its association with LTC residents’ malnutrition and low CC. Although overall diet quality using the MAR score was moderate, it was found to be associated with risk of malnutrition. Diet quality of several individual nutrients was also associated with malnutrition, while magnesium appears to be potentially relevant for CC. This research adds to our understanding of the importance of considering and improving micronutrient intake when attempting to prevent or treat malnutrition in LTC homes. Future work should be directed to ensuring nutrient dense menus, including micronutrient-enhanced foods, to support nutrient intake and potentially prevent malnutrition in LTC residents.

## Data Availability

The datasets generated and/or analyzed during the current study are currently not publicly available since they are still being used by the co-authors but are available from Heather Keller on reasonable request during the current study and will be available in 2020 for use by others.

## Abbreviations

CC: Calf circumference
CI: Confidence interval
CPS: Cognitive performance scale
DFE: Dietary folate equivalent
Ed-FED-Q: Edinburgh Feeding Evaluation in Dementia Questionnaire
IDDSI: International Dysphagia Diet Standardisation Initiative
IQR: Interquartile range
LTC: Long-term care
M3: Making the Most of Mealtimes
MAR: Mean adequacy ratio
MNA-SF: Mini-Nutritional Assessment-Short Form
MTD: Modified textured diets
NAR: Nutrient adequacy ratio
ONS: Oral nutritional supplement
PG-SGA: Patient-Generated Subjective Global Assessment
RAE: Retinol activity equivalents
SD: Standard deviation
